# Isolation and Nitrogen Removal Efficiency of the Heterotrophic Nitrifying-Aerobic Denitrifying Strain K17 From a Rare Earth Element Leaching Site

**DOI:** 10.3389/fmicb.2022.905409

**Published:** 2022-06-08

**Authors:** Jingang Hu, Xinyu Yang, Xiangyi Deng, Xuemei Liu, Junxia Yu, Ruan Chi, Chunqiao Xiao

**Affiliations:** Key Laboratory of Novel Biomass-Based Environmental and Energy Materials in Petroleum and Chemical Industry, Hubei Key Laboratory of Novel Reactor and Green Chemical Technology, Key Laboratory for Green Chemical Process of Ministry of Education, School of Environmental Ecology and Biological Engineering, Wuhan Institute of Technology, Wuhan, China

**Keywords:** heterotrophic nitrifying, aerobic denitrifying, *Pseudomonas mosselii* K17, nitrogen removal, rare earth ore

## Abstract

K17, an indigenous and heterotrophic nitrifying-aerobic denitrifying bacterium, was isolated from the soil of a weathered crust elution-deposited rare earth ore leaching site in Longnan County, China. Strain K17 was identified as *Pseudomonas mosselii*. In this study, the morphological characteristics of strain K17 were observed and the optimal ammonia nitrogen removal conditions for the strain were studied using a single-factor experiment. Key enzyme activities were determined, and we also explored the ammonia nitrogen removal process of strain K17 on simulated leaching liquor of the rare earth element leaching site. Based on the determination of ammonia nitrogen removal and enzyme activity, it was found that strain K17 has both heterotrophic nitrifying and aerobic denitrifying activities. In addition, single-factor experiments revealed that the most appropriate carbon source for strain K17 was sodium citrate with a C/N ratio of 10 and an initial NH_4_^+^-N concentration of 100 mg/l. Furthermore, the optimal initial pH and rotation speed were 7 and 165 r/min, respectively. Under optimal conditions, the ammonia nitrogen removal efficiency of strain K17 was greater than 95%. As an indigenous bacterium, strain K17 has great potential for treating residual ammonium leaching solutions from rare earth element leaching sites.

## Introduction

Rare earth elements are considered the ‘mother of new materials’ and are indispensable in high-tech fields, such as defence, medicine, clean energy and aerospace (Dutta et al., 2016; Li et al., 2020). The wide application of rare earth elements makes their replacement impossible. A new ion-adsorbed rare earth ore with stable chemical properties was discovered in Jiangxi Province, China in 1969 (Liu et al., 2021). In this type of ion-adsorbed rare earth deposit, leaching technology is generally used to exchange rare earth elements in a trivalent cation state with an ammonium sulphate aqueous solution (Liu et al., 2019). Unfortunately, a large amount of ammonia nitrogen accumulates in the mining soil due to leaching. The long-term slow release of residual ammonia nitrogen into the soil causes the concentration of ammonia nitrogen in the environment surrounding the mining area to exceed that of the standard for a long period. The accumulation of ammonia nitrogen contributes to multi-scale environmental concerns, such as water quality deterioration, vegetation destruction, human health hazards and biodiversity loss in aquatic systems (Rout et al., 2014, 2015, 2016). Therefore, ammonia nitrogen pollution is not conducive to the green and sustainable development of the rare earth industry. For that reason, the treatment of ammonia nitrogen pollution in mining areas is essential.

Compared with the high cost and low efficiency of other methods, for instance, electro-dialysis, ion exchange, ammonia volatilisation and reverse osmosis, biological denitrification is more promising because of its higher efficiency, operational simplicity and lower maintenance cost (Rout et al., 2017a,b). In the past few years, researchers have abandoned the complex treatment process of traditional biological nitrogen removal for the more promising simultaneous nitrification and denitrification (SND) process (Rout et al., 2017b). The biological removal of nitrogen involves a combination of nitrification and denitrification, and SND simultaneously completes these important steps (Zinatizadeh and Ghaytooli, 2015; Cao et al., 2017).

Since Wehrfritz et al. (1993) speculated the heterotrophic nitrifying-aerobic denitrifying (HN-AD) model (Li et al., 2015), some heterotrophic bacteria, such as *Pseudomonas stutzeri* (Zhang et al., 2011), *Bacillus* sp. (Kim et al., 2005), *Providencia rettgeri* (Patureau et al., 2001) and *Rhodococcus* sp. (Chen et al., 2012), have been found to have heterotrophic nitrifying-aerobic denitrifying function. The mentioned bacteria showed higher growth rates compared to autotrophs and a stronger tolerance to complex environments (Li et al., 2015). HN-AD was demonstrated using a single microbial consortium, with organic carbon as an electron donor as well as nitrite or nitrate as an electron acceptor (Rout et al., 2018). Some scholars have suggested that heterotrophic nitrifying-aerobic denitrifying has two main pathways. One involves complete nitrification and denitrification, in which ammonium is oxidised to nitrite and nitrate, in turn, and then denitrified (Huang et al., 2013). The other involves denitrification through intermediate hydroxylamine instead of nitrate and nitrite (Zhao et al., 2010, 2012). However, because the enzyme activity levels are extremely low and the intermediate products may not be easily detected, the process involved is unclear and requires further exploration (Ren et al., 2014).

The isolated strains are generally found in places, such as river sediments, landfill leachate and lake sludge, but less often in rare earth mines. Furthermore, because of the low pH and high concentration of sulphates in mine drainage, the mine soil environment is relatively poor (Bonnail et al., 2017). Compared with foreign strains, indigenous microorganisms may be more tolerant to the harsh environment of mines and the ammonia nitrogen removal efficiency of wastewater may be better. An indigenous, HN-AD strain, K17, was isolated from the soil of a weathered crust elution-deposited rare earth ore leaching site in Longnan County, Ganzhou, Jiangxi Province, China. We explored the ammonia nitrogen removal ability of strain K17 under different conditions (carbon source, C/N, rotation speed and pH) and explored the enzyme activity of strain K17 to explore its ammonia nitrogen removal pathway. Simultaneously, the entire ammonia nitrogen removal process was simulated, thereby providing a theoretical basis for practical applications.

## Materials and Methods

### Isolation and Identification of Heterotrophic Nitrifying Bacteria

The soil samples for this experiment were obtained from a rare earth element leaching site in an ion-type rare earth mine in Longnan County, Ganzhou, Jiangxi Province in China. A 10 g soil sample was added to a conical flask filled with 100 ml sterile water and vibrated at 30°C and 165 rpm for 30 min. After shaking for 15 min, the supernatant was collected to obtain a suspension of soil samples. A 50 ml volume of the cell suspension was added to 100 ml of HN-AD medium (containing (NH_4_)_2_SO_4_ 0.4714 g, sodium citrate 5 g, NaCl 2 g, MgSO_4_·7H_2_O 0.5 g, MnSO_4_·4H_2_O 0.01 g, K_2_HPO_4_ 1.0 g, FeSO_4_·7H_2_O 0.04 g and distilled water 1,000 ml, pH 7.0–7.5) and incubated at 30°C and 165 rpm for 48 h. Thereafter, 10 ml of bacterial suspension was added to 100 ml of fresh culture medium in a 250 ml flask for enrichment cultivation of bacterial cultures at 30°C and 165 rpm for 2 days. This process was repeated thrice. The isolation and purification of bacterial strains was achieved using planarisation lines. Thereafter, the pure samples were selected and individually detected for their nitrogen removal capability. The HN-AD strain with the highest NH_4_^+^-N removal efficiency was used in subsequent tests.

### Morphological Observation and 16S rRNA Gene Sequencing of Strain K17

Strain morphology was observed by scanning electron microscopy (SEM). The strain with strongest ammonia nitrogen removal ability was sent to Shanghai Majorbio Bio-Pharm Technology Co., Ltd. for sequencing using general primers 27F (5′-AGAGTTTGATCCTGGCTCAG-3′) and 1492R (5-GGTTACCTTGTTACGACTT-3′). The sequencing results were compared using the NCBI database,^1^ and a phylogenetic tree was constructed by MEGA 6.0 using the neighbour-joining method for analysis.

### Growth and Ammonia Nitrogen Removal Characteristics of Strain K17

50 ml of HN-AD medium with an initial ammonia nitrogen concentration of 100 mg/l was inoculated with a 2% bacterial solution and cultured in a conical flask in order to observe the growth of *Pseudomonas mosselii* K17. Meanwhile, a control group which had ammonia nitrogen without *P. mosselii* K17 was set. In the process of culturing, the pH, temperature and rotation speed were maintained at 7, 30°C and 165 rpm, respectively. Samples were aliquoted from the medium every 2 h to measure the OD_600_, and the concentrations of NH_4_^+^-N, NO_3_^−^-N and NO_2_^−^-N.

### Effect of Culturing Conditions on Growth and Ammonia Nitrogen Removal of Strain K17

The ammonia nitrogen removal characteristics of the strain K17 under different culture conditions (different carbon sources, C/N ratios, pH values and rotation speeds) were studied. Glucose, sucrose, sodium acetate, potassium sodium tartrate and soluble starch were each used as the only carbon source rather than sodium citrate in the carbon source experiments. The effects of the C/N ratio on nitrogen removal efficiency were investigated by adjusting the C/N ratio to 4, 6, 8, 10, 12 and 16. The effects of pH were determined by adjusting the initial pH to 4, 5, 6, 7, 8, 9 and 10 by adding 1 mol/l HCl or NaOH. The dissolved oxygen (DO) concentration was varied by adjusting the rotation speed to 0, 55, 110, 165 and 220 rpm to test the effect of DO on the nitrogen removal efficiency. Only a single factor was adjusted at a time in the experimental design; aside from these factors, the medium had a constant ammonium concentration of 100 mg/l and was cultured at pH 7.0, 30°C, C/N of 10 and 165 rpm for 12 h. OD_600_ and ammonium nitrogen concentrations were determined by taking samples from shake flasks at regular intervals.

### Enzyme Assay

Strain K17, designated for extract preparation, was collected from the medium (after 12 h of cultivation) by centrifugation, washed three times in a potassium phosphate buffer (PPB; 0.01 mol/l, pH 7.4) and re-suspended in the centrifuge tube. After crushing with an ultrasonic cell grinder, the cell-free extract (crude enzyme solution) was obtained *via* centrifugation at 4°C for 10 min at 10000 rpm. Coomassie Brilliant Blue was used to determine the protein concentration in the cell-free extract, thereby determining the specific activity of the enzymes. The oxidation of ammonium chloride, reduction of nitrate to nitrite and reduction of NH_2_OH and nitrite in the reaction mixture were used as indicators in the determination of ammonia monooxygenase (AMO), nitrate reductase (NR), hydroxylamine oxidase (HAO) and nitrite reductase (NIR; Zhao et al., 2010). Four reaction mixtures I (AMO), II (NR), III (NIR) and IV (HAO) were prepared. Reaction mixture I (5 ml) contained enzyme extract, 0.2 mm NADH and 10 mm Tris–HCl buffer (pH 7.4), and the reaction was initiated by adding NH_4_Cl (Ge, 2017). Reaction mixture II (5 ml) contained enzyme extract, 10 mm PPB (pH 7.4) and 0.2 mm NADH, and the reaction was initiated by adding NaNO_3_ (Zhao et al., 2010). Reaction mixture III (5 ml) contained 0.2 mm NADH, enzyme extract and 10 mm PPB (pH 7.4), and the reaction was initiated by adding NaNO_2_ (Zhao et al., 2010). Reaction mixture IV (5 ml) contained the enzyme extract, 0.001 mm K_3_[Fe (CN)_6_], 0.004 mm EDTA and 10 mm Tris–HCl buffer (pH 7.4), and the reaction was initiated by adding NH_2_OH (Ge, 2017). The amount of enzyme that catalysed the conversion of 1 μmol of substrate per minute is 1 unit of enzyme activity (U). The specific activity (U/mg) was calculated by dividing the number of enzyme units by the protein mass (mg).

### Removal of Ammonia Nitrogen With Different Concentrations by Strain K17

Based on the HN-AD medium, the simulated leaching solution of residual ammonium in the rare earth element leaching site was cultivated under an initial ammonia concentration (200, 300, 400, 500, 600, 700 and 800 mg/l) by controlling a single variable. The K17 seed solution (OD_600_ ≈ 1) was inoculated into HN-AD medium at 2% inoculum volume and cultured at 30°C and 165 rpm. Samples were collected every 12 h to detect the mass concentration of OD_600_ and NH_4_^+^-N in the culture medium in the preliminary examination of strain K17 for its ability to de-nitrify residual ammonium leaching solution from rare earth element leaching sites under different ammonia nitrogen concentrations.

### Analytical Methods

The growth of the strain was determined by measuring the absorbance at 600 nm. The concentration of NH_4_^+^-N was detected by Nessler’s reagent spectrophotometry (SEPA, 2002). The concentration of NO_3_^−^-N was determined by ultraviolet spectrophotometry (SEPA, 2002). The concentration of NO_2_^−^-N was measured at 540 nm using N-(1-naphthalene)-diaminoethane spectrophotometry (SEPA, 2002). The hydroxylamine content was analysed by UV–VIS spectrophotometry. Furthermore, the pH was measured using a Mettler SevenCompact pH metre (S210). The morphological characteristics of the strain were observed using SEM. Cell fragmentation was performed using an ultrasonic cell crusher.

### Statistical Analysis

The results are the arithmetic mean values of three independent experiments and all experimental results are expressed as the arithmetic mean ± standard deviation (SD). All data were subjected to one-way ANOVA by Tukey’s HSD test using SPSS software (version 19.0), and the level of significance was set at *p* < 0.05. Experimental data figures were obtained using the OriginPro 9.0.

## Results and Discussion

### Identification of Strain K17

**Figure 1:**
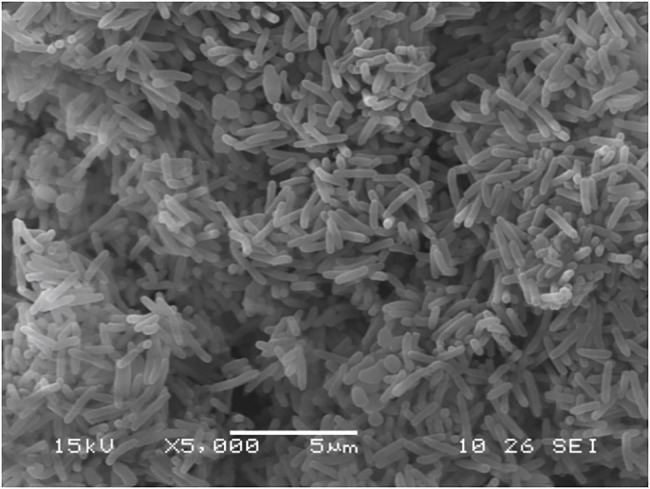
The morphology of *Pseudomonas mosselii* K17. Observed by scanning electron microscope (5,000×).

**Figure 2:**
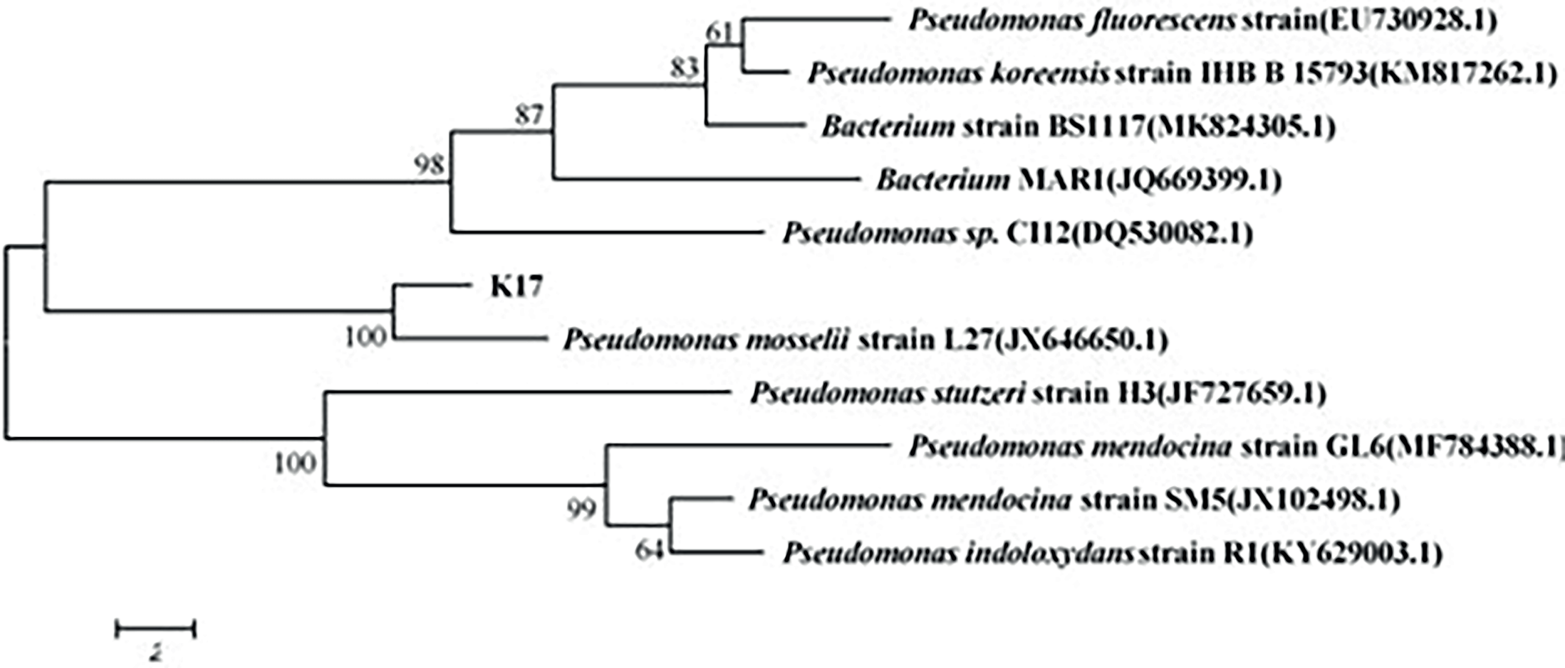
The phylogenetic tree of strain K17. According to the comparison result of GenBank database, it was constructed by neighbour-joining method using MEGA6.0 software.

Six strains were isolated and purified; among these, strain K17 had the strongest ammonia nitrogen removal ability. Strain K17 was gram-negative, its colony on the plate had a yellow-brown colour with a smooth surface and regular edges. Using a scanning electron microscope (JSM-5510LV), the cells of strain K17 were observed as short rods (Figure 1). *Via* gene sequencing, a 1,449-bp 16S rRNA fragment was obtained. The 16S rRNA homology sequence alignment was performed in the GenBank database, and it was found that the sequence was very similar to *Pseudomonas*. The phylogenetic tree was constructed by neighbour-joining method using MEGA6.0 software (Figure 2). The phylogenetic tree showed that strain K17 and *P. mosselii* strain L27 belonged to the same group, having a similarity of more than 99%. Based on morphological identification and 16S rRNA gene sequencing, strain K17 was identified as *P. mosselii*. The accession number of the 16S rRNA sequence of strain K17 in the GenBank database is MW547500.

### Growth and Ammonia Nitrogen Removal Characteristics of Strain K17

**Figure 3:**
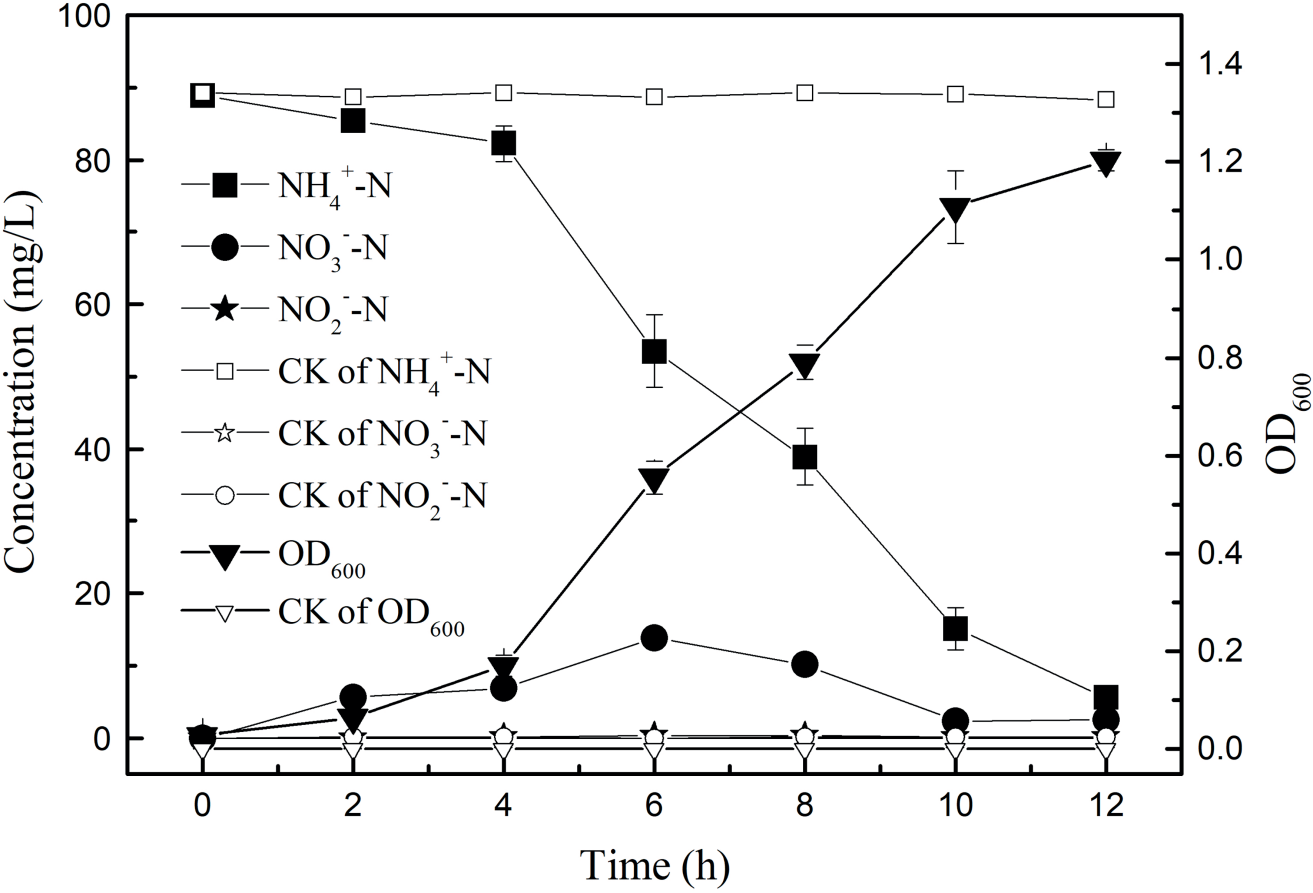
Growth curve and heterotrophic nitrifying characteristics of strain K17. The mixture was cultured at 165 rpm and 30°C for 12 h.

In the medium with (NH_4_)_2_SO_4_ as the only nitrogen source, the growth and ammonia nitrogen removal of strain K17 were shown in Figure 3. The results showed that the removal efficiency of ammonia nitrogen was significantly correlated with the growth of strain K17 (*p* < 0.01). During the logarithmic growth period (4–12 h), the strain K17 grew rapidly, and the OD_600_ was 1.20 at 12 h. At the same time, ammonia nitrogen was quickly removed, and the rate of ammonia nitrogen removal by strain K17 reached 9.57 mg/l/h. This removal rate was better than that of *Pseudomonas tolaasii* Y-11 (2.04 mg/l/h; He et al., 2016), *Acinetobacter* sp. Y16 (0.092 mg/l/h; Huang et al., 2013) and *Bacillus subtilis* A1 (3.52 mg/l/h; Yang et al., 2011). Some researchers have reported that HN-AD bacteria can oxidise ammonium to nitrate or nitrite and convert these products to N_2_O and/or N_2_ by denitrification (Zhao et al., 2012; Huang et al., 2013; Sun Z. et al., 2016). Therefore, NO_2_^−^-N and NO_3_^−^-N accumulated in the culture process but were gradually consumed after rapid growth. In this study, the concentration of NO_3_^−^-N in the culture medium first increased and then decreased, before finally accumulating to a certain amount. In the process of removing NH_4_^+^-N by *Acinetobacter* sp. T1, NO_3_^−^-N had a similar change trend (Chen et al., 2019). A small amount of NO_2_^−^-N accumulated in the medium during the reaction, which may be because NO_2_^−^-N is an intermediate product. It is inferred that the dissimilatory ammonia nitrogen removal pathway of strain K17 may be$\;\mathrm{NH}4+-N\to {\mathrm{NH}}_{2}\mathrm{OH}\to {\mathrm{NO}}_{2}^{-}-N⇌{\mathrm{NO}}_{3}^{-}-N$, leaving the NO_3_^−^-N (or NO_2_^−^-N) to be reduced to gaseous nitrogen. However, some HN-AD bacteria, such as *Cupriavidus* sp. S1 (Sun Z. et al., 2016), *Acinetobacter* sp. (Yang et al., 2019) and *Acinetobacter junii* (Yang et al., 2015), did not detect (or was very low levels) NO_3_^−^-N, NO_2_^−^-N during ammonia removal. *Pseudomonas aeruginosa* DBT1BNH3 grew well in the presence of nitrate, but no nitrite was detected (Velusamy and Krishnani, 2014). Their ammonia dissimilation pathway may be to convert NH_4_^+^-N to hydroxylamine and then to gaseous nitrogen. Velusamy and Krishnani (2013) found that *Alcaligenes faecalis* used O_2_ or NO_2_ as an electron acceptor to oxysome hydroxyl amine to N_2_O. Studies have shown that the HN-AD bacteria have multiple nitrogen metabolism pathways, giving it a greater advantage in the process of wastewater denitrification. At the same time, more research needs to be done on the denitrification mechanism of HN-AD bacteria.

### Single-Factor Experiments to Study the Factors Influencing Growth and Ammonia Nitrogen Removal Performance

#### Effect of Carbon Sources on Growth and Ammonia Nitrogen Removal Efficiency

**Figure 4:**
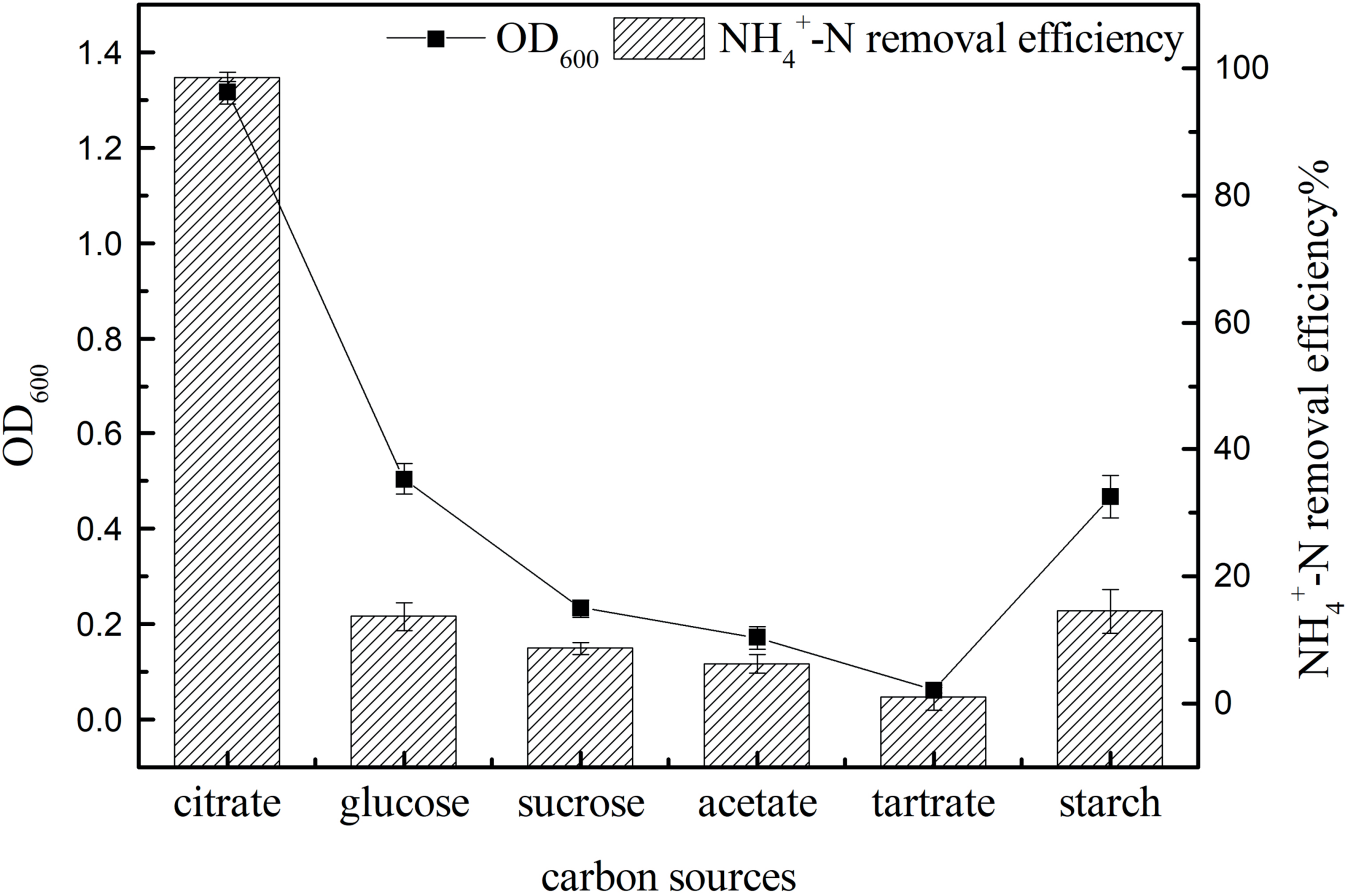
Effect of carbon source on the growth and ammonia nitrogen removal efficiency of strain K17. The mixture was cultured at 165 rpm and 30°C for 12 h.

Carbon sources can provide electron donors for the growth of microorganisms and are an important factor affecting bacterial growth and nitrogen removal (Chen et al., 2015). In the shake flask culture, the growth and ammonia nitrogen removal characteristics of strain K17 with different carbon sources were studied. As shown in Figure 4, carbon sources had a significant effect on the growth and ammonia nitrogen removal of strain K17 (*p* < 0.05). This may be because bacteria have different utilisation rates of carbon sources with different molecular weights and molecular structures, so that they show different growth and ammonia nitrogen removal characteristics under different carbon source conditions (Sun Q. et al., 2016; Chen et al., 2019). The most suitable carbon source for the growth and ammonia nitrogen removal of strain K17 was sodium citrate. By now the OD_600_ value was 1.315 ± 0.024 and the NH_4_^+^-N removal efficiency was 98.57 ± 0.69%. Sodium citrate might be the most ideal carbon source because it could directly enter the metabolic pathway without modification, which made the medium more alkaline during nitrification and was beneficial to the nitrification process (Brierley and Wood, 2001). In the next experiments, sodium citrate was used as the most ideal exogenous carbon source for the growth and ammonia nitrogen removal of strain K17.

#### Effect of C/N Ratio on Growth and Ammonia Nitrogen Removal Efficiency

**Figure 5:**
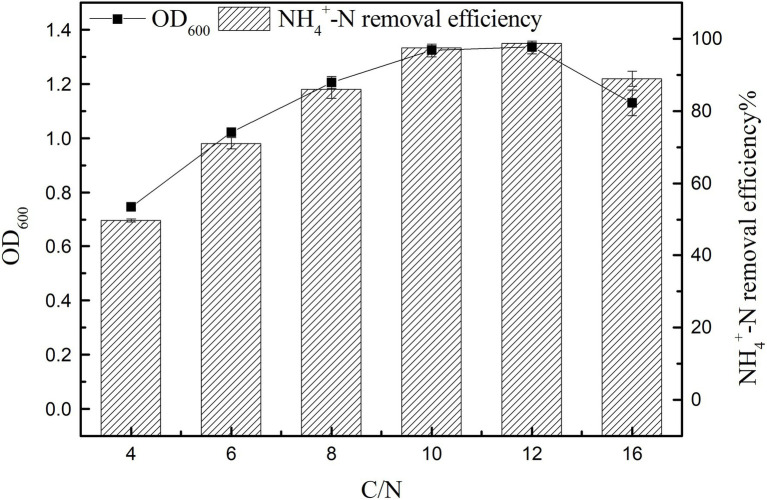
Effect of C/N ratio on the growth and ammonia nitrogen removal efficiency of strain K17. The mixture was cultured at 165 rpm and 30°C for 12 h.

The results of strain K17 cultured at different C/N ratios for 12 h were shown in Figure 5. There were significant differences in the growth and ammonia nitrogen removal of strain K17 under different C/N ratios (*p* < 0.05). When C/N ratio was 12, maximum nitrogen consumption and OD_600_ were 98.66 ± 0.38% and 1.335 ± 0.024, respectively. Notably, with a further increase in C/N, the NH_4_^+^-N removal efficiency decreased. And the growth and NH_4_^+^-N removal efficiency were low when the C/N ratio was 4 and 6. At low C/N ratios, the low removal rate of ammonia nitrogen is mainly due to an insufficient carbon supply, which inhibits the growth of microorganisms and reduces the availability of electron donors for ammonia nitrogen removal (Huang et al., 2013). The results showed that a high C/N ratio inhibits bacterial growth and ammonia nitrogen removal. It is possible that sources with high amounts of organic carbon inhibited the action of enzymes involved in denitrification. The nitrogen removal rate reached 97.52 ± 0.50% when the C/N ratio was 10 and, in terms of comprehensive economic benefits, the suitable C/N ratio of strain K17 was 10.

#### Effect of Rotation Speed on Growth and Ammonia Nitrogen Removal Efficiency

**Figure 6:**
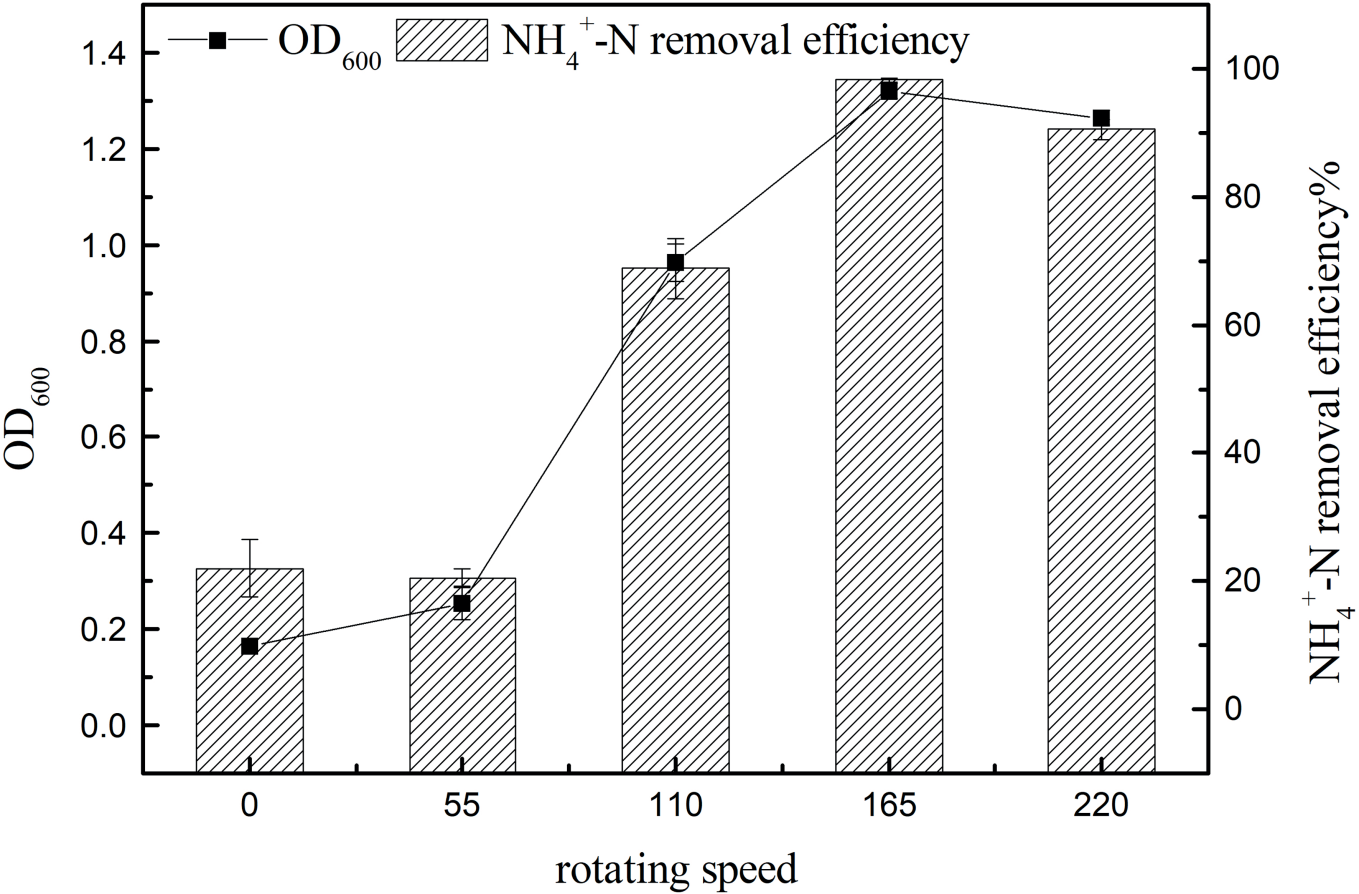
Effect of rotation speed on the growth and ammonia nitrogen removal efficiency of strain K17. The mixture was cultured at 165 rpm and 30°C for 12 h.

The main control parameters of SND, such as dissolved oxygen (DO), can be affected by changing the rotation speed (Hocaoglu et al., 2011). Figure 6 shows that with the increase in rotation speed, ammonia nitrogen removal and cell growth were significantly promoted (*p* < 0.05), and the maximum value of ammonia nitrogen removal was 98.25 ± 0.34% at 165 rpm (Figure 6). The small difference in rotation speed between 0 and 55 rpm may be due to the small difference in dissolved oxygen in the culture medium under low rotation speeds. The growth of strains at 0 rpm may be because of the residual dissolved oxygen in the culture medium and oxygen in the air. When the rotation speed reached 220 rpm, the growth and ammonia nitrogen removal of strain K17 were lower than that at 165 rpm. This might be because a high concentration of dissolved oxygen inhibits bacterial growth or because the rotation speed is too high and produces a large shearing force that damages the cells. Therefore, the most suitable rotation speed for the growth and ammonia nitrogen removal of strain K17 was approximately 165 rpm.

#### Effect of Initial pH on Growth and Ammonia Nitrogen Removal Efficiency

**Figure 7:**
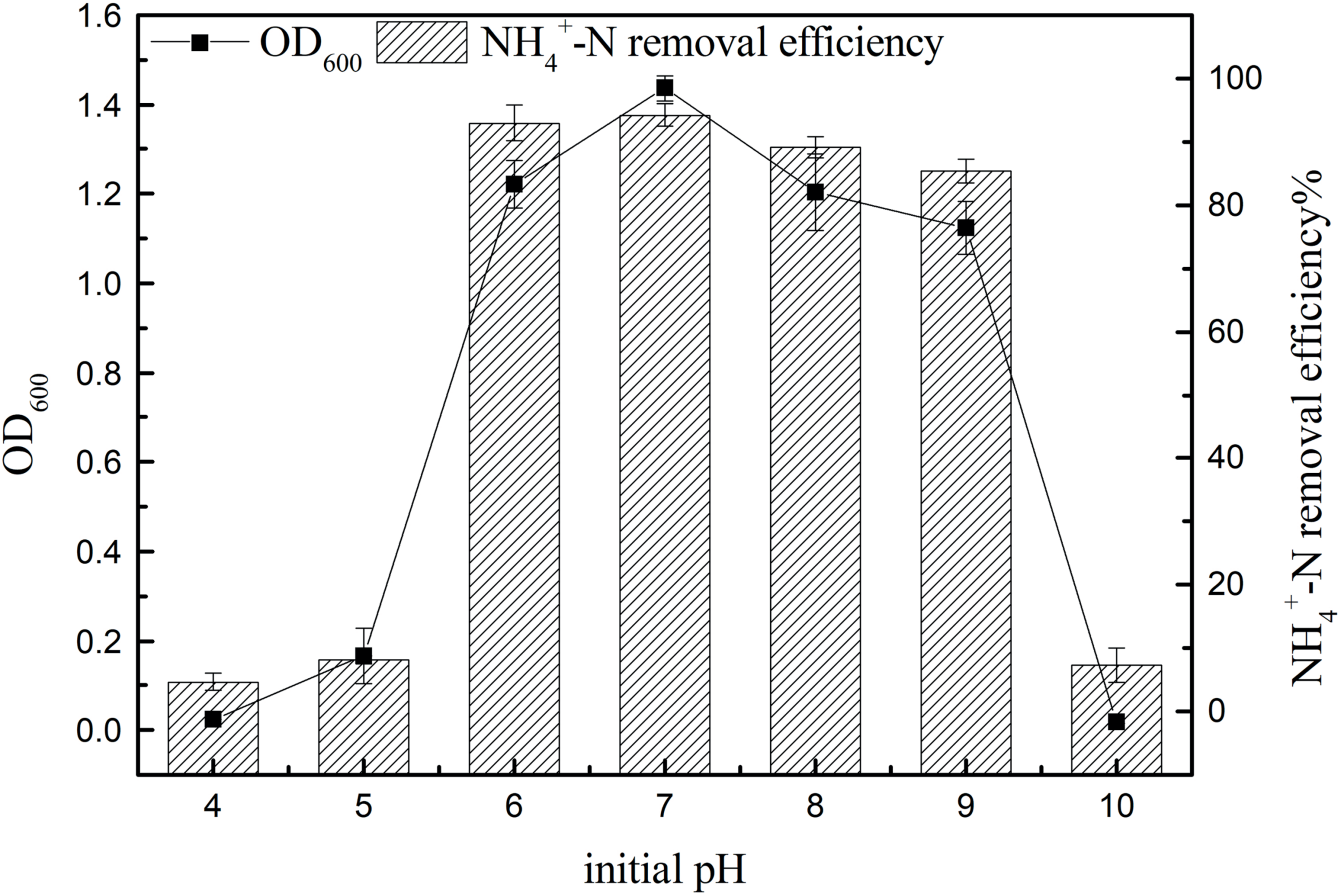
Effect of initial pH on the growth and ammonia nitrogen removal efficiency of strain K17. The initial ammonia nitrogen concentration was 100 mg/l, and the mixture was cultured at 165 rpm and 30°C for 12 h.

The cell growth and the ammonia nitrogen removal performance of K17 at different pH values are shown in Figure 7. The results showed that the initial pH had a significant effect on nitrogen removal efficiency (*p* < 0.05). The NH_4_^+^-N removal efficiency was consistent with cell growth, and K17 performed well in the pH range of 6–9, with the removal rate of ammonia nitrogen being more than 85%. The ammonia nitrogen removal rate and OD_600_ were highest at an initial pH of 7 after 12 h of cultivation, and the NH_4_^+^-N removal rate reached 94.23 ± 1.84%. Generally, severely acidic and alkaline conditions are inhibitory to the growth of K17, with very little increase in OD_600_. In a mildly alkaline environment, the removal efficiency of ammonia nitrogen was better, which may be because of the high content of free ammonia which favours the enzyme AMO (Ren et al., 2014). Strain K17 can grow in an acidic environment and maintain a certain nitrogen removal rate, which may be due to the long-term existence of strain K17 in the acidic soil of the mining area and its adaptability to acidic environments. Strain K17 has the ability to remove ammonia nitrogen over a wide pH range, which has great application potential in the treatment of acidic wastewater.

### Enzyme Assay

The enzymatic activities of AMO, HAO, NR and NIR were measured to verify the ammonia oxidation pathway of strain K17 (Table 1). High activity levels of AMO (0.155 ± 0.009 U) and HAO (0.037 ± 0.004 U) in the heterotrophic nitrifying pathway were detected. Some researchers believe that the production mechanism of nitrogen-containing gas does not only include the denitrification of nitrate and nitrite, but also the production of intermediate hydroxylamine instead of nitrate and nitrite (Joo et al., 2005). However, the activities of NR and NIR in the denitrification pathway were detected, and it was speculated that the nitrogen-containing gas of strain K17 was produced by the denitrification of nitrate and nitrite rather than by the production of intermediate hydroxylamine. In addition, some scholars have proposed that in the presence of nitrate, NR in *Pseudomonas* sp. could be induced in the process of ammonium oxidation (Zhao et al., 2012). Meanwhile, the HN-AD mechanism of the isolates could be further investigated by identifying the denitrification function genes. Velusamy and Krishnani (2013, 2014) confirmed that the HN-AD strains had an aerobic denitrification system by identifying some denitrification functional genes, such as nitrite reductase (nirS), nitric oxide reductase (qnorB) and nitrous oxide reductase (nosZ) genes.

**Table 1:** Enzyme activity and specific activity.

Enzyme type	Enzyme activity/U	Specific activity/U•mg^−1^
AMO	0.1546 ± 0.0087	0.2654 ± 0.0149
HAO	0.0374 ± 0.0042	0.0643 ± 0.0073
NR	0.0170 ± 0.0066	0.0292 ± 0.0112
NIR	0.0094 ± 0.0007	0.0161 ± 0.0011

*Data are represented by the mean of three replicates ± standard deviations. p < 0.05*.

### Ammonia Nitrogen Removal Performance of Strain K17

**Figure 8:**
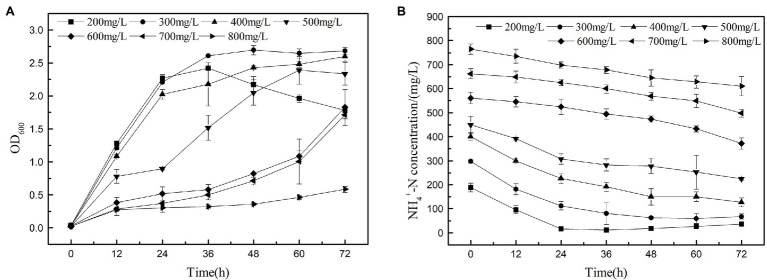
Nitrification performance of strain K17 cultured under different ammonia nitrogen conditions. The initial ammonia nitrogen concentration was 200, 300, 400, 500, 600, 700 and 800 mg/l. The mixture was cultured at 165 rpm and 30°C for 12 h. **(A)** Growth characteristics of strain K17 under different ammonia nitrogen concentrations. **(B)** Ammonia nitrogen removal characteristics of strain K17 under different ammonia nitrogen concentrations.

The heterotrophic nitrifying of K17 at ammonium concentrations of 200, 300, 400, 500, 600, 700, and 800 mg/l is shown in Figure 8. The results show that strain K17 has a higher ammonia nitrogen removal rate when the ammonia nitrogen concentration is lower than 300 mg/l. Strain K17 can grow and remove nitrogen at a higher ammonia nitrogen concentration (700 mg/l), but high concentrations of ammonia nitrogen inhibited the growth and ammonia nitrogen removal ability of strain K17. When the initial ammonia nitrogen concentration was 800 mg/l, strain K17 hardly grew. The maximum removal rates under low and medium NH_4_^+^-N and loads were significantly higher than those seen with *A. faecalis* No. 4 (Joo et al., 2005). According to the report, another heterotrophic nitrifier *P. rettgeri* YL have the highest tolerance of up to 300 mg/l NH_4_^+^-N, and the ammonia tolerance of strain K17 was much higher than that of it (Taylor et al., 2009). The study found that the ammonia nitrogen concentration first decreased and then increased because of the decay of bacteria when the ammonia nitrogen concentration was lower than 300 mg/l. Therefore, when using strain K17, one should keenly control the ammonia nitrogen removal time in the actual application process. When the initial ammonia nitrogen concentration was 800 mg/l, the growth of strain K17 was 0.58, but the ammonia nitrogen concentration decreased by 152.70 mg/l. This might be due to the long-term shake flask culture being under high ammonia nitrogen concentration, which volatilised the culture medium part of the ammonia nitrogen. It also explained why strain K17 had almost no growth at the beginning, but the OD_600_ value was 0.58 at 72 h, which further explained why the bacteria could tolerate a concentration of 700 mg/l ammonia nitrogen. Therefore, the high tolerance of ammonia nitrogen makes strain K17 a suitable prospect in the treatment of ammonia nitrogen wastewater.

## Conclusion

*Pseudomonas mosselii* K17 is a bacterial strain isolated from the soil of a rare earth element leaching site. It can exhibit high efficiency in heterotrophic nitrifying-aerobic denitrifying under a broad range of ammonia concentrations. The most appropriate conditions for heterotrophic nitrifying of *P. mosselii* K17 included sodium citrate as the carbon source as well as a C/N ratio of 10, pH of 7.0 and shaking conditions of 165 rpm. The enzyme activity results indicated that the nitrogen metabolism pathway of the strain might be due to the coupling of heterotrophic nitrifying and aerobic denitrifying. In addition, strain K17 can treat wastewater with a broad range of concentrations of ammonia nitrogen. In conclusion, as an indigenous bacterium, strain K17 has great application potential in treating residual ammonium leaching solutions from rare earth element leaching sites.

## Data Availability Statement

The datasets presented in this study can be found in online repositories. The names of the repository/repositories and accession number(s) can be found at: https://www.ncbi.nlm.nih.gov/genbank/, MW547500.

## Author Contributions

JH, XY, XD, and XL designed the study, conducted the experiments, and analysed the results of the experiments. CX, JY, and RC provided financial support and guidance on the ideas for the study and provided significant help in conducting the experiments. CX, XD, XL, and RC provided critical advice on the interpretation of the data. CX and RC supervised the study. All authors contributed to the article and approved the submitted version.

## Funding

This study was supported by the National Key Research and Development Program of China (2018YFC1801802) and the Innovative Team Program of Natural Science Foundation of Hubei Province (2021CFA032).

## Conflict of Interest

The authors declare that the research was conducted in the absence of any commercial or financial relationships that could be construed as a potential conflict of interest.

## Publisher’s Note

All claims expressed in this article are solely those of the authors and do not necessarily represent those of their affiliated organizations, or those of the publisher, the editors and the reviewers. Any product that may be evaluated in this article, or claim that may be made by its manufacturer, is not guaranteed or endorsed by the publisher.

## Footnotes

^1^ https://www.ncbi.nlm.nih.gov/
